# Mayfly response to different stress types in small and mid-sized lowland rivers

**DOI:** 10.3897/zookeys.980.54805

**Published:** 2020-10-28

**Authors:** Marina Vilenica, Mladen Kerovec, Ivana Pozojević, Zlatko Mihaljević

**Affiliations:** 1 University of Zagreb, Faculty of Teacher Education, Trg Matice Hrvatske 12, Petrinja, Croatia University of Zagreb Petrinja Croatia; 2 University of Zagreb, Faculty of Science, Department of Biology, Rooseveltov Trg 6, Zagreb, Croatia University of Zagreb Zagreb Croatia

**Keywords:** Environmental stress, Ephemeroptera, feeding guilds, longitudinal zonal associations, pollution

## Abstract

Freshwater ecosystems are endangered worldwide by various human pressures, resulting in dramatic habitat and species loss. Many aquatic invertebrates respond to disturbances in their habitat, and mayflies are among the most sensitive ones. Therefore, we investigated mayfly response to anthropogenic disturbances at 46 study sites encompassing slightly to heavily modified small and mid-sized lowland streams and rivers. Mayfly nymphs were sampled between April and September 2016 using a benthos hand net. A total of 21 species was recorded, with *Cloeon dipterum* (Linnaeus, 1761) being the most frequently recorded one. Nevertheless, the taxa richness was rather low per site, i.e., between zero and nine. Assemblage structure had a high share of lower reaches and lentic (potamic and littoral) elements, and detritivores (gatherers/collectors and active filter feeders). This indicates that hydromorphological alterations lead to assemblage “potamisation” in small and mid-sized rivers. More mayfly species were related to higher oxygen concentration and lower water temperature, abundance of aquatic vegetation and total organic carbon. Additionally, the assemblage diversity and abundance were negatively associated with increasing intensive agriculture area at the catchment scale. This study confirms mayfly bio-indicative properties, i.e., their sensitivity to alterations of their habitat and pollution, but also provides new data related to mayfly response to the impacted environment. Those data can be used for management and protection activities of lowland rivers and their biota according to the requirements of the European Water Framework Directive.

## Introduction

Freshwater ecosystems represent an indispensable resource of water supplies for humans (Carpenter et al. 2011), but they also have a crucial role in biodiversity maintenance and conservation (Previšić et al. 2009; Ivković and Plant 2015). Therefore, it is essential they remain in good ecological status (Dudgeon et al. 2006; Vörösmarty et al. 2010). Nevertheless, the status of many aquatic systems is far from good worldwide (Carpenter et al. 2011). Various anthropogenic impacts represent major threats to aquatic biodiversity and make lotic habitats among the most endangered ones (Malmqvist and Rundle 2002; Hering et al. 2006; Stoddard et al. 2006). Human population growth, increased urbanisation and industrialisation have led to increased demands for land use for purposes of agriculture, forestry, irrigation activities and wetland drainage, resulting in alterations of habitat morphology, hydrological regime and causing degraded water quality, pollution and increased sediment erosion into lotic systems (Waters 1995; Dudgeon et al. 2006; Woodward et al. 2012). By altering their natural condition, such activities largely downgrade the habitat integrity, which results in reduced ecological function and biodiversity (Steffen et al. 2015), including native species loss (Carpenter et al. 2011). The habitat characteristics change dramatically: formation of macrophyte assemblages is disturbed (Jones et al. 2014; Turunen et al. 2017), habitat heterogeneity and availability for macroinvertebrates is reduced (Jones et al. 2012), while primary production (Louhi et al. 2017) and decomposition of organic matter (Lecerf and Richardson 2010) are highly altered.

As freshwater organisms live almost continuously in the aquatic environment, they clearly respond to all those environmental stresses (Morse et al. 2007; Vilenica et al. 2019; 2020). The aquatic assemblages can respond to alterations of their habitats with their structure differing from a reference state, i.e., they can show characteristics of “rhithralisation” (e.g., caused by channel straightening) or “potamisation” (e.g., caused by the impounding) (Jungwirth et al. 2000; Moog and Chovanec 2000; Kokavec et al. 2018; Vilenica et al. 2016; 2019), or there is a change in the trophic structure (Brasil et al. 2013). By observing the assemblages’ structural alterations, we can conclude that the lotic system has been altered, which in the end indicates a certain level of ecological disturbance (Moog 2002; Vilenica et al. 2016). Mayflies are able to colonise all kinds of freshwater habitats but are found to be the most diverse in lotic ones. They are among particularly sensitive aquatic macroinvertebrates, mainly disappearing when faced even with small-scale disturbance in their habitat (Firmiano et al. 2017; Vilenica et al. 2019). Previous studies demonstrated that the majority of species can tolerate a rather narrow range of environmental factors, being highly sensitive to oxygen depletion, acidification, and various contaminants such as metals, ammonia, nitrogen, phosphorous (Moog et al. 1997; Vilenica et al. 2017, 2019). Therefore, the absence/presence of a particular species can tell us a lot about the quality of the environment it inhabits. Ecological assessments in different regions worldwide, as well as at habitats of various ecological status are necessary for effective conservation and management of freshwater habitats and their biota (Hughes et al. 1986; Stoddard et al. 2008). Therefore, in order to obtain additional data on mayfly response to anthropogenic disturbances in their habitat, we investigated mayfly assemblages and their relationship with environmental factors at 46 slightly to heavily modified lotic habitats.

## Materials and methods

### Study area

**Figure 1.**
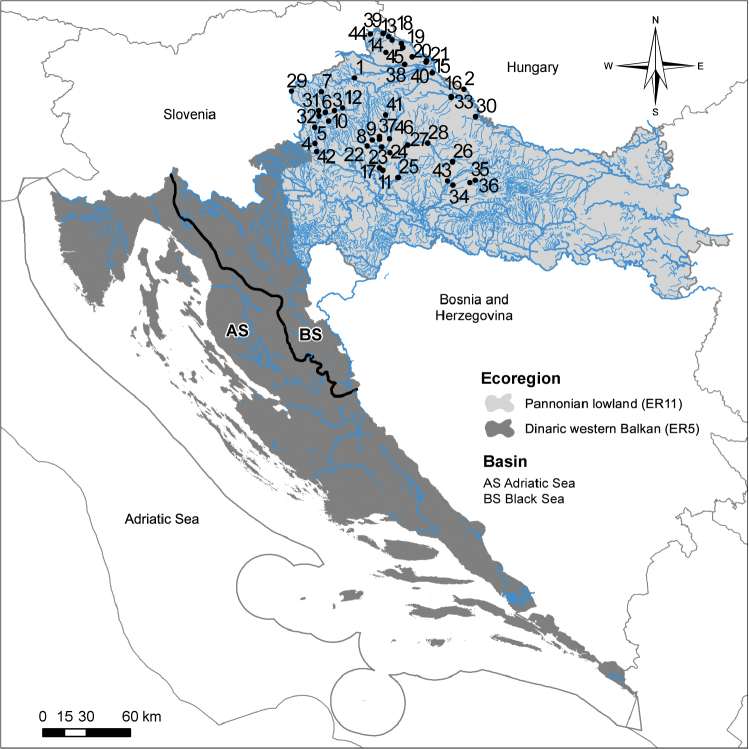
Map of the 46 study sites located in the Pannonian lowland ecoregion in Croatia. *Legend: Study sites: **1** Bednja, Stažnjevec village **2** Ždalica, Ždala village **3** Krapina, Bedekovčina village **4** Krapina, Zaprešić town **5** Krapina, Kupljenovo village **6** Krapinica, Zabok town **7** Krapinica, Krapina town **8** Rajna, between Vrbovec town and Lonjica village **9** Zlenin, Vrbovec village **10** Vukšinac, Stubice village **11** Deanovac lateral canal, near Ivanić Grad town **12** Reka, Lovrečan village **13** Brodec, Peklenica village **14** Lateral canal Mihovljan, Čakovec town **15** Poloj, between Legrad and Đelekovec villages **16** Zdelja, Molve village **17** Lonja, near Ivanić Grad town **18** Jalšovnica, Ferketinec village **19** Bošćak, Domašinec village **20** Bistrec, Rakovnica I **21** Bistrec, Rakovnica II **22** Zelina, Božjakovina village **23** Connecting canal Zelina-Lonja-Glogovnica-Česma, Poljanski lug village **24** Glogovnica, before mouth to Česma **25** Česma, Obedišće village **26** Česma, Pavlovac village **27** Česma, Sišćani village **28** Česma, Narta village **29** Sutla, Luke Poljanske village **30** Rogostrug, Podravske Sesvete village **31** Kosteljina, Jalšje village **32** Horvatska, Veliko Trgovišće village **33** Bistra Koprivnička, Molve village **34** Toplica, Sokolovac village **35** Toplica, downstream from Daruvar town **36** Toplica, upstream from Daruvar town **37** Luka, Vrbovec town **38** Sewage collector, Prelog town **39** Gornji potok, between Selnica and Praporčan villages **40** Kotoribski kanal, Kotoriba village **41** Črnec, Gornji Dubovec vilage **42** Gostiraj, Ježdovec village **43** Tomašica, Tomašica village **44** Jalšovec, between Bukovje and Štrigova villages **45** Murščak, between Domašinec and Stara Straža villages **46** Glogovnica, Koritna village.

The study encompassed 46 lotic slow-flowing study sites (Tables 1, 2, Fig. 1), including heavily modified streams and rivers (by, for instance, channelling and/or modification of the water flow or riverbed, removal of the riparian vegetation and pollution). The majority of the study sites are located in the vicinity of agricultural areas or cattle farms. Sampling was conducted between April and September 2016. Within the research, it was not possible to include a reference site. True reference sites are not available due to long-lasting and strong anthropogenic influence. The relatively high ratio of urban areas and even more agricultural ones are present in their catchment. The majority of the rivers have been channelled for agricultural land use purposes, or have limited lateral movement because of dykes protecting urban areas and settlements. During RFI (River Fauna Index) and assessment system development, the best available sites were chosen. The reference RFI and metrics value was calculated by adding 20% of the metric range to the high/good boundary. Study sites are part of the national monitoring program. From 25 m (small streams) to 50 m (mid-sized rivers) long sampling area was selected to cover the greatest possible diversity of microhabitats representative of the reach.

**Table 1.** List of the 46 degraded lowland streams and rivers investigated in Croatia, with environmental parameters measured at the time of macroinvertebrate sampling. Codes of the study sites are as in Fig. 1. Legend: River size – S – small rivers (catchment area less than 100 km^2^), M – medium-sized rivers (catchment area less than 1000 km^2^). Channel width and water depth are expressed in meters. HYMO Group in SIMPER analysis – according to RFIEQR (1 – good and high; 2 – moderate; 3 – poor and bad). Tw – water temperature (°C), Oxy – dissolved oxygen content (mg/L), Con – conductivity (μS/cm), pH – pH, dominant substrates – lithal – stones, gravel; fine sediment – silt, mud, sand; phytal – aquatic vegetation.

Study site	River size	Width	Depth	HYMO Group	Coordinates (N/E)		Tw	Oxy	Con	pH	Dominant substrates
1	S	6.0	1.5	1	46.24	16.17	14	10.09	503	7.96	Lithal, fine sediment, phytal
2	S	3.0	0.8	1	46.17	17.15	19	3.64	718	7.54	Fine sediment, phytal
3	S	8.0	1.0	1	46.04	15.99	13	9.96	556	8.15	Fine sediment, phytal
4	M	18.0	2.0	3	45.83	15.82	16	9.02	605	8.16	Fine sediment, phytal
5	M	16.0	30.0	3	45.93	15.82	16	8.05	628	8.13	Lithal, fine sediment, phytal
6	M	8.0	1.0	3	46.03	15.91	19	8.77	710	8.05	Lithal, fine sediment
7	S	6.0	0.4	1	46.15	15.88	13	8.97	574	8.17	Lithal, fine sediment, phytal
8	S	5.0	0.5	2	45.86	16.33	16	8.20	796	8.50	Fine sediment, phytal
9	S	3.0	0.3	2	45.86	16.40	15	3.92	702	7.64	Fine sediment, phytal
10	S	5.0	0.4	2	45.98	15.94	17	10.20	484	8.15	Fine sediment
11	S	4.0	0.6	3	45.67	16.42	11	6.02	564	7.85	Fine sediment, phytal
12	S	3.0	0.4	1	46.05	16.07	13	10.25	545	8.47	Lithal, fine sediment
13	S	2.5	0.5	2	46.50	16.47	16	9.81	446	7.82	Fine sediment, phytal
14	S	1.5	0.3	1	46.40	16.45	14	7.81	316	7.60	Lithal, phytal
15	S	3.0	0.5	1	46.27	16.86	21	6.90	982	7.52	Fine sediment, phytal
16	S	1.5	0.3	1	46.12	17.03	25	9.20	885	9.20	Fine sediment, phytal
17	S	6.0	1.0	1	45.69	16.39	11	8.12	625	8.12	Fine sediment, phytal
18	S	2.0	0.5	1	46.48	16.51	14	9.55	332	7.58	Fine sediment, phytal
19	S	5.0	0.8	2	46.43	16.60	16	7.50	391	7.48	Fine sediment, phytal
20	S	4.0	1.0	2	46.37	16.69	16	9.89	735	8.19	Fine sediment, phytal
21	S	7.0	1.0	1	46.34	16.81	17	8.80	608	8.18	Fine sediment, phytal
22	M	4.0	0.8	3	45.82	16.28	14	8.41	592	8.22	Lithal (phytal sporadically)
23	M	10.0	0.8	3	45.81	16.41	12	10.70	616	8.48	Fine sediment, phytal
24	M	15.0	2.0	3	45.78	16.49	11	6.60	610	7.98	Lithal, fine sediment
25	M	10.0	1.0	3	45.63	16.56	12	5.75	581	8.02	Fine sediment, phytal
26	M	6.0	1.0	3	45.72	17.04	21	3.58	429	7.52	Fine sediment, phytal
27	M	12.0	1.2	3	45.83	16.64	25	5.05	396	7.70	Lithal, phytal
28	M	14.0	1.0	3	45.84	16.82	23	6.75	401	7.78	Fine sediment, phytal
29	M	9.0	0.6	3	46.16	15.61	16	8.29	553	8.16	Fine sediment
30	M	6.0	1.5	2	46.00	17.25	17	9.17	551	7.77	Fine sediment, phytal
31	M	5.0	1.5	3	46.04	15.85	21	8.85	713	7.97	Fine sediment, phytal
32	M	2.5	0.4	3	46.00	15.86	19	8.78	732	8.02	Fine sediment, phytal
33	M	10.0	1.5	3	46.12	17.03	20	7.87	588	7.62	Fine sediment, phytal
34	M	4.0	0.5	3	45.58	17.04	24	8.30	461	8.04	Fine sediment
35	S	5.0	0.5	1	45.59	17.19	22	6.52	539	7.65	Fine sediment, phytal
36	S	4.5	0.3	1	45.61	17.24	18	8.95	465	8.23	Lithal, fine sediment
37	S	1.5	0.2	1	45.88	16.39	22	8.93	207	8.15	Fine sediment
38	S	2.0	0.2	1	46.32	16.62	12	5.22	524	7.52	Fine sediment
39	S	1.5	1.0	1	46.52	16.43	16	8.29	629	7.77	Lithal, fine sediment, phytal
40	S	3.5	0.6	2	46.34	16.82	17	5.70	574	5.68	Phytal
41	S	2.0	0.4	2	46.01	16.45	25	1.53	619	7.85	Fine sediment, phytal
42	S	2.5	0.3	1	45.78	15.84	20	6.90	670	7.85	Fine sediment, phytal
43	S	2.5	0.3	2	45.60	16.99	20	4.52	601	7.75	Lithal, fine sediment
44	S	2.0	0.5	1	46.51	16.31	12	8.80	740	8.45	Lithal, fine sediment
45	S	3.0	0.7	2	46.45	16.59	14	3.50	541	7.36	Phytal
46	M	-	-	3	45.87	16.49	9	6.68	578	7.77	Fine sediment, phytal

The study area is located in the Croatian part of the Pannonian lowland ecoregion (ER11) (Illies 1978). The area is characterised with temperate humid climate with warm summer (Cfb, Köppen classification) where the average temperature of the warmest month is below 22 °C (Šegota and Filipčić 2003). The average annual air temperature is around 12 °C and average annual rainfall is between 800 and 1100 mm (Zaninović et al. 2008).

### Sampling protocol

Mayfly nymphs were collected together with other macroinvertebrates (AQEM protocol- AQEM expert consortium 2002). At each site, 20 subsamples were collected proportionally according to available microhabitat presence, using a benthos hand net (25 × 25 cm; mesh size = 500 μm) and pooled into one composite sample. The substrates were mainly composed of fine sediment (sand, silt, mud), lithal (stones, gravel), and aquatic vegetation (submerged and emergent). Samples were stored in 96% alcohol and analysed in the lab.

In the laboratory, subsampling was done to reduce the effort for sorting and identification. At least 1/6 of the sample was sorted until the minimum targeted number of 700 individuals was reached. The rest of the sample was also inspected searching for macroinvertebrates which are not part of subsample analysed. Mayflies were identified to the lowest possible taxonomical level (very juvenile and/or damaged individuals were identified only to the genus or family level) using Müller-Liebenau (1969), Malzacher (1984) and Bauernfeind and Humpesch (2001). All voucher specimens are deposited at the Department of Biology, Faculty of Science, University of Zagreb, Croatia.

### Environmental factors

At each study site, the following environmental parameters were measured at the time of macroinvertebrate sampling: water temperature, dissolved oxygen concentration (using the oximeter WTW Oxi 330/SET), conductivity (with the conductivity meter WTW LF 330), pH (using the pH-meter WTW ph 330), mean channel width and maximum water depth (using a hand meter on approximately 100 meter long reach of specific site) (Table 1). The remaining environmental parameters are presented as the mean value of 12 composite samples collected over a one-year period (January – December 2016) (Table 2). Water chemistry analyses were carried out according to standard methods (Author2007). Land use variables were defined from the share of land use categories at the catchment scale, extracted from Corine Land Cover (CLC) data (CLC Hrvatska 2013) using ArcGIS version 10.2.1 (Esri Corp., Redlands, CA, USA). A relative measure of hydromorphological (HYMO) alternation was given by calculating the River fauna index (RFI) using macroinvertebrate species sensitivity scores. A version of the RFI adapted for Croatian rivers and streams following Urbanič (2014) gives a score of HYMO alternation based on the response of macroinvertebrate assemblages. The scores are then normalised with regard to reference states in the form of the WFD (Water framework directive) recommended EQRs (ecological quality ratios) and range from 0 (the worst HYMO conditions) to 1 (reflecting reference states). The HYMO evaluation of rivers has been performed by European Standards EN 14614 and EN 15843. Type specific RFI was used as a relative measure of HYMO alternation because HYMO evaluations for all of the investigated rivers are not available.

**Table 2.** List of the 46 degraded lowland streams and rivers investigated in Croatia, with environmental parameters presented as mean value of 12 composite samples collected over a one-year period (January–December 2016) (including standard deviation, SD). Codes of the study sites are as in Fig. 1. Legend: NH_4_^+^ – ammonium (mgN/L), NO_3_- – nitrates (mgN/L), TN – total nitrogen (mgN/L), PO_4_^3−^ – orthophosphates (mgP/L), TOC – total organic carbon (mg/L), BOD_5_ – biological oxygen demand (mgO_2_/L), COD_Mn_ – chemical oxygen demand (mgO_2_/L).

Study site	NH_4_^+^	NO^3-^	TN	PO_4_^3-^	TOC	BOD_5_	COD_Mn_
	mean/SD	mean/SD	mean/SD	mean/SD	mean/SD	mean/SD	mean/SD
1	0.373/0.199	1.090/0.282	1.940/0.454	0.062/0.030	4.037/0.531	2.308/0.915	3.942/1.033
2	0.014/0.008	0.100/0.077	0.466/0.115	0.016/0.016	5.235/2.197	2.531/2.294	4.463/2.286
3	0.224/0.142	1.033/0.235	1.788/0.332	0.094/0.053	3.429/0.952	2.192/0.960	3.567/1.120
4	0.178/0.237	1.284/0.438	1.928/0.473	0.050/0.035	3.671/1.026	1.767/1.314	3.733/1.700
5	0.316/0.268	1.227/0.323	2.073/0.449	0.065/0.037	3.671/1.002	2.150/1.218	3.933/1.522
6	0.437/0.221	1.392/0.294	2.443/0.496	<0.025	4.292/1.456	4.969/1.585	6.636/1.624
7	0.920/0.556	1.179/0.202	2.95/0.950	<0.025	4.917/1.575	6.663/1.021	8.878/2.742
8	0.460/0.533	0.948/0.793	1.772/1.328	0.333/0.137	6.297/1.451	3.153/1.441	8.469/1.714
9	3.240/3.931	1.110/0.977	6.379/6.053	1.952/2.799	9.971/4.071	3.028/1.937	10.308/2.030
10	0.061/0.036	1.038/0.424	1.517/0.799	<0.025	3.292/1.515	4.039/2.120	5.772/3.010
11	0.333/0.413	1.701/1.479	2.527/2.185	0.215/0.091	7.608/1.688	3.019/1.209	7.992/2.235
12	0.256/0.169	1.365/0.570	2.032/0.552	<0.025	2.233/0.463	3.395/0.460	4.822/1.926
13	0.279/0.112	2.600/0.553	3.775/0.758	0.046/0.058	1.874/0.569	1.525/0.652	1.898/0.894
14	1.599/1.792	2.304/1.205	5.025/2.419	0.340/0.269	5.582/1.108	2.683/0.878	4.694/2.138
15	0.158/0.188	0.540/0.286	1.080/0.130	0.058/0.057	4.561/1.100	1.880/1.132	5.070/1.630
16	0.513/1.238	1.416/0.853	2.880/1.608	0.141/0.141	5.922/4.891	1.650/1.297	5.147/4.094
17	0.306/0.264	1.852/1.071	2.370/1.135	0.310/1.148	7.352/1.694	3.240/1.087	8.449/2.717
18	0.018/0.005	3.918/0.865	5.250/1.091	0.010/0.008	2.095/0.777	1.033/0.473	2.085/1.088
19	0.064/0.103	1.317/2.013	1.694/3.011	0.020/0.022	1.333/4.571	1.317/0.709	1.338/0.543
20	0.276/0.781	6.541/1.196	8.192/1.483	0.080/0.207	<1.000/0.760	1.146/1.175	0.936/0.495
21	0.053/0.096	3.478/0.721	4.683/1.069	0.018/0.021	1.237/0.365	1.183/0.629	1.097/0.507
22	0.157/0.153	1.873/0.717	2.260/0.861	0.136/0.056	3.633/1.140	2.303/0.630	4.584/1.491
23	0.373/0.379	1.700/0.728	2.370/1.001	0.343/0.183	5.388/1.283	2.998/1.118	6.672/2.563
24	0.574/0.494	1.938/1.099	3.702/1.599	0.333/0.303	5.489/2.591	5.600/3.252	10.933/4.000
25	0.487/0.232	1.503/0.881	2.744/0.978	0.171/0.116	8.458/2.809	3.895/0.698	10.949/5.439
26	0.067/0.099	0.796/0.221	1.170/0.446	0.105/0.063	6.230/3.294	4.178/2.417	11.689/5.089
27	0.514/0.537	1.965/1.346	3.799/2.105	0.208/0.094	7.134/3.248	8.033/3.588	14.122/5.349
28	0.096/0.055	1.285/0.850	2.168/1.227	0.103/0.043	6.046/2.803	4.967/3.297	12.633/4.379
29	0.141/0.121	1.071/0.258	1.713/0.376	0.077/0.036	3.868/0.989	1.517/0.536	4.117/0.920
30	0.115/0.123	0.654/0.409	1.375/0.270	0.040/0.065	3.096/0.757	1.242/0.575	2.320/1.134
31	0.493/0.378	0.952/0.411	1.968/0.577	<0.025	3.761/0.987	4.683/1.356	6.483/1.925
32	0.198/0.156	1.031/0.429	1.733/0.457	<0.025	4.672/1.354	3.949/2.710	5.772/3.876
33	0.223/0.207	0.668/0.394	1.392/0.545	0.225/0.211	2.993/1.132	1.200/0.544	2.472/1.001
34	0.413/0.432	2.179/0.427	3.227/0.784	0.224/0.180	3.316/1.727	4.089/3.240	6.678/1.281
35	0.818/0.422	1.283/0.255	3.067/0.836	0.224/0.117	3.518/2.219	7.133/4.520	8.722/4.855
36	<0.015/0.000	1.070/0.157	1.000/0.439	0.035/0.022	2.184/1.759	4.963/11.023	4.850/3.754
37	0.211/0.262	3.127/1.100	3.615/1.260	6.545/3.751	7.596/1.717	2.734/1.460	8.558/1.782
38	1.919/0.962	0.967/0.804	3.758/1.141	0.131/0.149	2.023/0.977	3.058/1.561	1.443/0.795
39	0.537/1.151	1.251/0.699	2.567/1.315	0.092/0.187	3.866/1.503	2.208/1.308	3.451/1.431
40	4.093/3.559	0.554/0.432	5.033/3.206	0.248/0.308	4.695/1.898	4.042/1.254	3.568/2.977
41	5.007/9.111	1.484/0.903	9.567/10.991	1.569/0.850	9.585/4.024	6.225/1.299	15.489/7.189
42	1.240/1.059	2.915/1.127	5.168/1.728	0.387/0.124	4.146/0.727	4.626/1.106	5.897/1.183
43	3.495/2.977	3.880/5.995	14.023/10.061	1.488/1.851	8.142/4.419	22.856/27.457	18.933/8.407
44	0.320/0.520	0.931/0.568	1.858/0.678	0.069/0.063	3.651/0.975	1.500/0.729	3.224/1.660
45	0.103/0.190	5.545/1.319	7.258/1.939	0.025/0.031	2.120/0.298	0.729/0.378	1.807/0.802
46	1.220/1.098	1.996/0.912	3.970/2.179	0.322/0.154	5.082/1.524	4.366/1.171	6.112/1.952

### Data analysis

Mayfly assemblages from sites classified as high and good by the RFIEQR (EQR > 0.6) represented Group 1, from sites classified as moderate (0.4 < EQR <0.6) represented Group 2 and from sites classified as poor and bad (EQR < 0.4) represented Group 3 in the analysis of similarity percentages (SIMPER) of the (Bray-Curtis) similarity (Clarke 1993) between mayfly assemblages. This was done in order to determine how mayfly assemblages differ among sites of different degrees in HYMO alternation in terms of species composition and abundance contribution.

The composition of mayfly assemblages in terms of the trophic structure and longitudinal zonal associations of species at each study site was analysed using the classification given by Buffagni et al. (2009; 2020), while the methodology was described in Vilenica et al. (2018). Study sites without mayfly records, and sites with one taxon where we could not identify the specimens to the species level (i.e., sites 17 and 18) were excluded from the analysis.

In order to ordinate mayfly occurrence with respect to environmental variables, the Canonical Correspondence Analysis (CCA) was used. The analysis was performed using data for 21 taxa (rare species were downweighed) and 14 environmental variables. The Monte Carlo permutation test with 499 permutations was used to test the statistical significance of the relationship between all taxa and all variables.

Mayfly taxa abundances were correlated against agricultural land cover data, using the Spearman coefficient, in order to determine if and to what extent does this type of land cover in the catchment area influence specific taxa occurrence. Mayfly species richness, abundance and local diversity (Shannon index) were plotted against the ratio of intensive agriculture in the catchment in order to determine the “general” mayfly response in relation to increased agricultural pressures.

The Bray-Curtis similarity index, Shannon diversity index and SIMPER analyses were conducted in Primer 6 (Clarke and Gorley 2006). The CCA analysis was performed using CANOCO 5.00 (ter Braak and Šmilauer 2012). Mayfly/intensive agriculture graphs were plotted, and regression equations were calculated and tested for significance using Statistica 13.0 (TIBCO Software Inc. 2017). The species data were log-transformed prior to analyses. All figures were processed with Adobe Illustrator CS6.

## Results

### Mayfly assemblages

A total of 21 species (27 taxa) was recorded of which the most widespread was *Cloeon dipterum* (Linnaeus, 1761), recorded at 18 study sites, while *Serratella ignita* (Poda, 1761) was the most abundant (Table 3). Nine species were recorded at only one study site, with *Heptagenia flava* Rostock, 1878, *Alainites muticus* (Linnaeus, 1758), and *Oligoneuriella rhenana* (Imhoff, 1852) being the rarest ones (Table 3). The highest number of taxa was recorded at study sites 22 and 36 (nine), while no mayfly was recorded at sites 35, 37, 38, 41, 43, 45 (Table 3).

**Table 3.** Mayfly taxa recorded (individuals/m^2^) at the 46 degraded lowland streams and rivers investigated in Croatia. Codes of the study sites are as in Fig. 1.

Taxa codes	1	2	3	4	5	6	7	8	9	10	11	12	13	14	15	16	17	18	19	20	21	22	23	24	25	26	27	28	29	30	31	32	33	34	35	36	37	38	39	40	41	42	43	44	45	46
a	0	16	0	0	0	0	0	0	0	0	0	0	0	0	0	0	0	0	0	0	0	0	0	0	0	0	0	0	0	0	0	0	0	0	0	0	0	0	0	0	0	80	0	0	0	0
b	0	0	0	0	0	0	0	0	0	0	0	0	0	0	0	0	0	0	0	0	0	0	0	0	0	0	0	0	0	0	0	0	0	0	0	2	0	0	0	0	0	0	0	0	0	0
c	0	12	0	0	368	292	0	0	0	16	0	16	432	976	0	0	88	16	2224	330	906	16	0	8	0	438	68	158	64	276	108	544	1376	22	0	364	0	0	336	52	0	0	0	0	0	8
d	120	0	48	0	120	36	0	0	0	0	0	0	0	1008	0	0	0	0	0	2	316	0	0	0	0	16	168	17	0	0	172	96	408	12	0	0	0	0	0	8	0	0	0	0	0	0
e	0	0	720	0	80	252	16	0	0	0	0	112	0	80	0	0	0	0	0	0	0	0	0	0	0	0	292	1	328	0	0	0	0	12	0	98	0	0	16	0	0	0	0	0	0	0
f	0	0	0	0	0	0	0	0	0	0	0	0	0	0	0	0	0	0	0	0	0	0	0	0	0	0	0	0	0	0	0	0	0	0	0	4	0	0	0	0	0	0	0	0	0	0
g	0	0	16	0	0	0	0	0	0	0	0	16	0	0	0	0	0	0	0	0	0	0	0	0	0	0	0	0	0	0	0	0	0	0	0	206	0	0	0	0	0	0	0	0	0	0
h	0	0	0	0	0	0	16	0	0	0	0	0	128	0	0	0	0	0	104	16	142	0	0	0	0	0	0	0	0	80	0	32	24	0	0	0	0	0	832	0	0	0	0	224	0	8
i	0	0	0	0	0	0	0	0	0	0	0	0	0	0	0	0	0	0	0	0	18	80	0	0	16	0	0	0	0	0	0	0	0	0	0	6	0	0	0	0	0	0	0	0	0	0
j	0	48	0	148	0	0	0	32	64	0	128	0	0	32	4	960	0	0	0	0	0	0	24	0	160	44	0	59	0	0	154	480	360	4	0	0	0	0	0	8	0	128	0	0	0	0
k	0	0	0	72	288	0	0	0	0	0	0	0	0	0	0	0	0	0	0	0	0	64	0	0	0	0	0	68	0	0	2	0	0	0	0	0	0	0	0	4	0	0	0	0	0	0
l	0	0	0	0	0	0	0	0	0	0	0	0	0	0	0	0	0	0	0	0	0	16	16	0	0	0	0	0	0	0	0	0	0	0	0	0	0	0	0	0	0	0	0	0	0	0
m	24	0	144	0	24	0	32	0	0	144	0	1424	0	32	0	0	0	0	8	2	6	208	0	0	0	0	8	32	104	0	0	0	0	0	0	0	0	0	0	0	0	0	0	0	0	0
n	0	0	0	0	0	0	0	0	0	0	0	0	0	0	0	0	0	0	0	0	0	0	0	0	0	0	0	10	0	0	0	0	0	0	0	0	0	0	0	0	0	0	0	0	0	0
o	0	0	1984	0	0	0	48	0	0	144	0	1664	0	0	0	0	0	0	0	0	214	48	0	0	0	0	0	0	80	0	0	0	0	0	0	36	0	0	24	4	0	0	0	0	0	0
p	0	0	0	0	0	0	0	0	0	0	0	16	0	0	0	0	0	0	0	0	40	0	0	0	0	0	0	0	0	0	0	0	0	0	0	0	0	0	0	4	0	0	0	0	0	0
r	0	0	0	0	0	0	0	0	0	0	0	0	0	0	0	0	0	0	0	0	0	0	0	0	48	0	0	0	0	0	0	0	0	0	0	0	0	0	0	0	0	0	0	0	0	0
s	0	0	48	0	0	0	48	32	0	112	0	0	0	0	0	0	0	0	0	0	0	640	0	8	16	5	0	0	40	0	0	0	0	0	0	0	0	0	0	0	0	0	0	160	0	0
t	0	0	0	0	0	0	0	0	0	0	0	0	0	0	0	0	0	0	0	0	0	0	0	0	16	0	0	0	0	0	0	0	0	0	0	0	0	0	0	0	0	0	0	0	0	0
u	0	0	0	0	0	0	0	0	0	0	0	0	0	0	0	0	0	0	0	0	0	0	0	0	0	0	0	0	4	0	0	0	0	0	0	0	0	0	0	0	0	0	0	0	0	0
v	0	0	0	0	0	0	0	0	0	0	0	0	0	0	0	0	0	0	0	0	0	16	0	0	0	0	0	0	0	0	0	0	0	0	0	0	0	0	0	0	0	0	0	0	0	0
z	0	0	0	0	0	0	0	0	0	0	0	0	0	0	0	0	0	0	0	0	0	0	0	0	0	0	0	0	0	0	0	0	0	0	0	46	0	0	0	0	0	0	0	0	0	0
w	0	0	0	0	0	0	0	0	0	16	0	0	0	0	0	0	0	0	0	0	0	0	0	0	0	0	0	0	0	0	0	0	0	0	0	0	0	0	0	0	0	0	0	0	0	0
x	8	0	0	0	0	0	0	0	0	0	0	0	0	0	0	0	0	0	0	0	0	0	0	0	0	0	0	0	0	0	0	0	0	0	0	0	0	0	0	0	0	0	0	0	0	0
y	0	0	0	0	0	0	0	0	0	0	0	0	0	0	0	0	0	0	0	0	0	0	0	0	0	0	0	1	0	0	0	0	0	0	0	0	0	0	0	0	0	0	0	0	0	0
xx	0	0	0	0	0	0	0	0	0	16	0	0	0	0	0	0	0	0	0	0	0	16	0	0	64	0	0	0	0	0	0	0	0	0	0	0	0	0	0	0	0	0	0	0	0	0
xy	0	0	0	0	0	0	0	0	0	0	0	0	0	0	0	0	0	0	0	0	0	0	0	0	0	0	0	0	0	0	0	0	0	0	0	2	0	0	0	0	0	0	0	0	0	0

*Legend: a – juvenile/damaged Baetidae, b – *Alainites muticus* (Linnaeus, 1758), c – juvenile/damaged *Baetis* sp., d – *Baetis buceratus* Eaton, 1870, e – *Baetis fuscatus* (Linnaeus, 1761), f – *Baetis lutheri* Müller-Liebenau, 1967, g – *Baetis rhodani* (Pictet, 1843), h – *Baetis vernus* Curtis, 1834, i – *Centroptilum luteolum* Müller, 1776, j – *Cloeon dipterum* (Linnaeus, 1760), k – juvenile *Caenis* sp., l – *Caenis horaria* – (Linnaeus, 1758), m – *Caenis luctuosa* (Burmeister, 1839), n – *Caenis robusta* Eaton, 1884, o – *Serratella ignita* (Poda, 1761), p – *Ephemera danica* Müller, 1764, r – juvenile/damaged Leptophlebiidae, s – *Habrophlebia fusca* (Curtis, 1834), t – juvenile/damaged *Paraleptophlebia* sp., u – juvenile/damaged Heptageniidae, v – *Electrogena ujhelyii* (Sowa, 1981), z – juvenile *Ecdyonurus* sp., w – Ecdyonurus cf. macani Thomas & Sowa, 1970, x – *Ecdyonurus torrentis* Kimmins, 1942, y – *Heptagenia flava* Rostock, 1878, xx – *Potamanthus luteus* (Linnaeus, 1767), xy – *Oligoneuriella rhenana* (Imhoff, 1852).

The SIMPER group similarity analysis (Table 4) showed that all groups of sites were dominated by juvenile instars of *Baetis* sp. and had significant abundances of *Cloeon dipterum* present at most sites. *Baetis fuscatus* (Linnaeus, 1761) and *Baetis buceratus* Eaton, 1870 were associated with sites of both ends of the HYMO gradient (Group 1 and Group 3). Furthermore, *Baetis vernus* Curtis, 1834 individuals were associated with sites that had a lower degree of HYMO degradation (Group 1 and Group 2). Juvenile instars of *Caenis* sp. were usually associated with more degraded sites (Group 3), whereas *Serratella ignita* and *Caenis luctuosa* (Burmeister, 1839) were associated only with sites of good and high ecological status following the RFI.

**Table 4.** Results of the SIMPER analysis based on mayfly assemblages from sites of different hydromorphological (HYMO) alternation levels.

Species	Average abundance per site (ind/m^2^)	Similarity contribution within group (%)
Group 1 good and high EQR based on RFI (EQR > 0.6)		
Average similarity: 18.68		
*Baetis* sp. juv.	2.54	33.12
*Cloeon dipterum*	1.38	16.42
*Serratella ignita*	2.07	12.01
*Baetis fuscatus*	1.73	10.82
*Caenis luctuosa*	1.63	10.32
*Baetis vernus*	1.33	6.58
*Baetis buceratus*	1.16	3.59
Group 2 moderate EQR based on RFI (0.4 < EQR < 0.6)		
Average similarity: 31.33		
*Baetis* sp. juv.	4.00	58.12
*Baetis vernus*	2.09	21.63
*Cloeon dipterum*	1.23	12.51
Group 3 poor and bad EQR based on RFI (EQR < 0.4)		
Average similarity: 31.54		
*Baetis* sp. juv.	3.51	39.10
*Cloeon dipterum*	2.63	28.10
*Baetis buceratus*	2.21	16.08
*Baetis fuscatus*	1.45	5.57
*Caenis* sp. juv.	1.14	3.27

**Figure 2.**
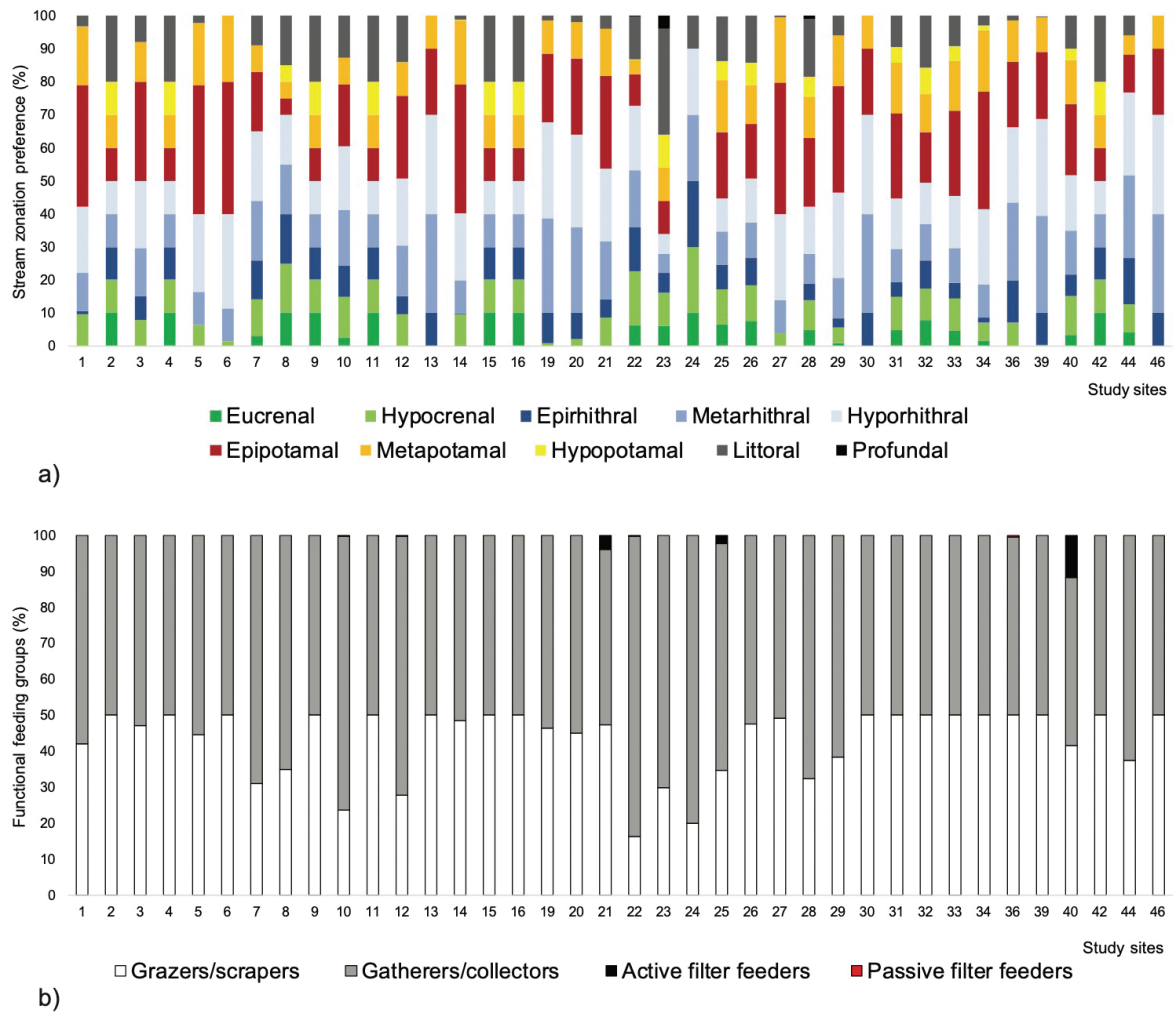
**a** Longitudinal zonal associations and **b** trophic structure of mayfly assemblages at the 46 degraded lowland streams and rivers investigated in Croatia. Study site codes are presented in Fig. 1.

Generally, a high share of lower reaches and lentic elements (potamic and littoral elements) was recorded: it was dominant (> 50 %) at 13 study sites, eight sites had an equal share of lower reaches/lentic and upper reaches elements (crenal and rhithral) (50:50 %), while16 study sites were dominated by upper reaches elements (> 50 %) (Fig. 2a). We also recorded a high share of detritivores (gatherers/collectors and active filter feeders): they were dominant at 21 study sites and equally represented as grazers/scrapers at the rest of the sites (Fig. 2b).

### Mayflies and environmental variables

**Figure 3.**
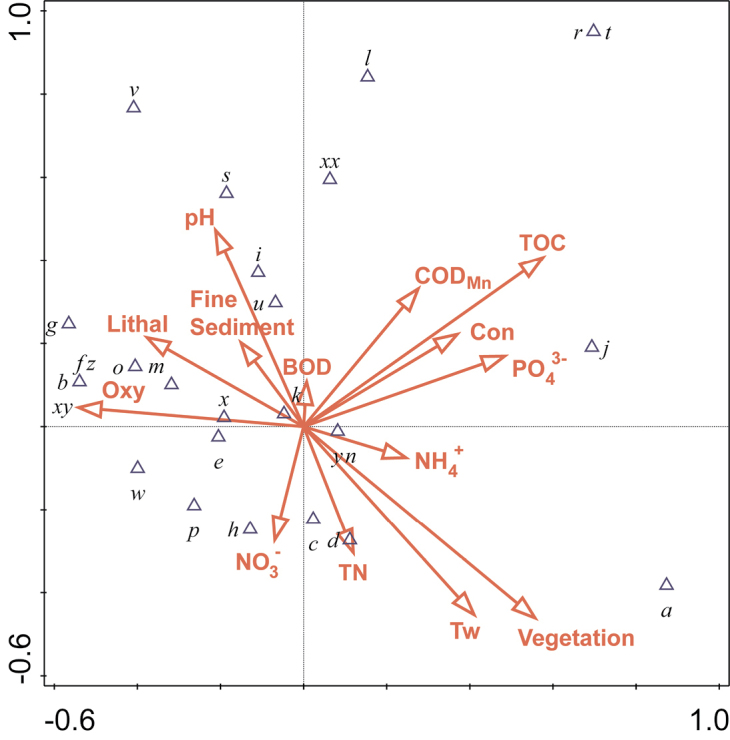
F1×F2 plane of the Canonical correspondence analysis (CCA) based on 21 mayfly taxa and 14 environmental variables. For the abbreviations of the taxa codes (blue triangle symbols) see Table 2. Legend: Environmental variables (red arrow symbols): Tw – water temperature (°C), Oxy – dissolved oxygen content (mg/L), Con – conductivity (μS/cm), pH – pH, NH_4_^+^ – ammonium (mgN/L), NO_3_- – nitrates (mgN/L), TN – total nitrogen (mgN/L), PO_4_^3−^ – orthophosphates (mgP/L), TOC – total organic carbon (mg/L), BOD_5_ – biological oxygen demand (mgO_2_/L), COD_Mn_ – chemical oxygen demand (mgO_2_/L), vegetation – aquatic vegetation/phytal, fine sediment – silt, mud and sand, lithal – stones and gravel.

The results of the ordination of species and environmental data of the CCA are presented on the F1 × F2 ordination plot (Fig. 3). The eigenvalues for the first two CCA axes were 0.40 and 0.25 and explained 50.9 % of the species-environment relations. The Monte Carlo permutation test showed that the species-environment ordination was significant (first axis: F-ratio = 4.23, p = 0.002; overall: trace = 1.28, F = 1.54, p = 0.006) indicating that mayfly assemblages were significantly related to the tested set of environmental variables. Axis 1 was related to total organic carbon (R = 0.49) and dissolved oxygen (R = -0.46), and axis 2 to aquatic vegetation (R = -0.37) and water temperature (R = -0.36), indicating that these were the most important parameters in explaining patterns of mayfly assemblages (Fig. 3).

**Figure 4.**
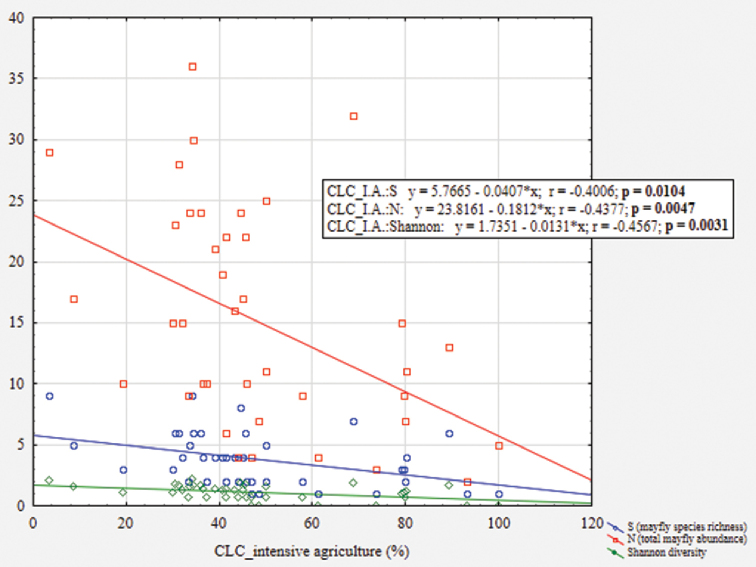
Scatterplot of mayfly species richness (S), abundance (N) and local diversity (Shannon index) against ratios of areas with intensive agriculture (CLC_I.A.) present in the catchment area of each study site.

Mayfly species richness, abundance and consequently also local diversity, were found to significantly decrease with increased ratios of intensive agriculture areas in the catchment area (Fig. 4).

Abundances of *Alainites muticus* (R = -0.303; p=0.041), *Baetis lutheri* Müller-Liebenau, 1967 (R = -0.303; p = 0.041), *Baetis rhodani* (Pictet, 1843) (R = -0.318; p = 0.031), *Oligoneuriella rhenana* (R = -0.303; p = 0.041) and juvenile instars of *Ecdyonurus* sp. (R = -0.303; p = 0.041) were found to significantly decrease with increased ratios of intensive agriculture area in the catchment area. Only taxa with statistically significant correlations are presented.

## Discussion

Our results indicate that a relatively high number of mayfly species can be found in anthropogenically impacted freshwater habitats. Nevertheless, at a large part of the study sites (i.e., 72 %) taxa richness was low, i.e., between zero and four taxa, corroborating previous studies (Vilenica et al. 2016; 2019). Mayflies inhabit both lotic and lentic habitats, although upper and middle reaches of fast-flowing streams, and ecologically intact large rivers harbour the highest mayfly diversity (Bauernfeind and Soldán 2012; Vilenica et al. 2016; 2018). Therefore, such low species richness, not typical for a lotic habitat (Bauernfeind and Moog 2000; Zedková et al. 2014; Vilenica et al. 2018), could be a consequence of various disturbances present at those sites, such as channelling, eutrophication, pollution, and microhabitat homogeneity (Axelsson et al. 2011; Carvalho et al. 2013; Ligeiro et al. 2013). In many cases, we observed shoreline erosion, as the emergent vegetation along the habitat edges, together with surrounding vegetation was mowed. This could have resulted in an increased input of sediments into the habitats, which could have influenced the habitat physico-chemical characteristics and hydrological cycle, resulting in reduced water quality and habitat heterogeneity (Mendes et al. 2017 and references herein). Consequently, these habitats showed to be less favourable for a high number of mayfly species. The majority of study sites were inhabited by widespread and generalist species (Popielarz and Neal 2007; Bauernfeind and Soldán 2012), yet sites with more microhabitat heterogeneity and higher water velocity, had also several microhabitat specialists, such as *Baetis lutheri* and *Ecdyonurus torrentis* for mesolithal, and *Centroptilum luteolum* as specialists for macrophytes (Buffagni et al. 2009; 2020).

The Zelina stream in Božjakovina (site 22) and Toplica River upstream from Daruvar town (site 36) showed somewhat higher species richness, yet their assemblages mainly consisted of species inhabiting a wide range of habitats, such as *Baetis rhodani*, *Centroptilum luteolum*, *Serratella ignita* and *Caenis luctuosa* (Buffagni et al. 2009; 2020; Bauernfeind and Soldán 2012). The most interesting finding was a record of *Oligoneuriella rhenana* at Toplica River, which is considered rare in Croatia (Vilenica et al. 2015; 2018). Although the species can tolerate some variations of environmental factors, its presence indicates that the ecological condition of Toplica River upstream from Daruvar town is not as poor as at the majority of other sites (Găldean 1999; Petrovici and Tudorancea 2000). Another interesting species was the rarest in our study, a riverine *Heptagenia flava*, uncommon in Croatian waters (Vilenica et al. 2015). Although the species was reported to have rather high ecological plasticity, usually it does not inhabit heavily polluted rivers (Vidinova and Russev 1997). Therefore, the species record at Česma River in Narta (site 28) could be considered as an accidental finding, as shown by Vidinova and Russev (1997). On the other hand, two eurytopic and euryvalent species (i.e., with wide tolerance towards the environmental conditions and habitat type), *Cloeon dipterum* and *Serratella ignita*, were recorded as the most common and the most numerous, respectively (Buffagni et al. 2009; 2020; Bauernfeind and Soldán 2012; Vilenica et al. 2019). Nevertheless, while discussing the total species richness at a particular site, we need to keep in mind that standardised sampling methods generally do not include sampling of underrepresented microhabitats, which could be important for some rare species (Haase et al. 2008). Therefore, in order to obtain a more complete species list, it might be beneficial to complement standardised quantitative sampling with a qualitative one.

Stream channelling is a widely used engineering practice designed for flood control and wetland draining, which affects the majority of hydrogeomorphological characteristics and processes at the channelled habitat. Due to these changes, the biota is also severely affected (Hupp 1992), i.e., the community structure and composition are changed and poorer (Waters 1995). Our results showed that mayfly assemblages have mainly consisted of taxa of potamic (lower reaches) and lentic preferences (e.g., *Baetis buceratus*, *Caenis horaria*) or wide range (e.g., *Cloeon dipterum*, *Centroptilum luteolum*, *Serratella ignita*) habitat type preferences (Buffagni et al. 2009; 2020; Bauernfeind and Soldán 2012). Moreover, *Baetis vernus*, *Caenis luctuosa* and *Serratella ignita*, species with relatively strong rhithral affinity (Biss et al. 2002) were predominantly associated with hydromorphologically less degraded sites, while species with more prominent potamic preference, such as *Baetis buceratus* and *Baetis fuscatus* (Schöll et al. 2005) were present at sites both with low and high degree of hydromorphological degradation. Some study sites showed a higher share of rhithral elements, yet that was mainly due to the dominance of eurytopic *Cloeon dipterum* (Buffagni et al. 2009; 2020). As the majority of sites are characterised by low microhabitat diversity, a high level of sedimentation and nutrients, assemblages were dominated by detritivores (Buffagni et al. 2009, 2020).

Previous researches showed that mayflies are highly dependent on specific environmental cues, and many species rapidly disappear when faced with anthropogenic disturbances in their habitat (Bauernfeind and Moog 2000; Goulart and Callisto 2005; Stepanian et al. 2020). Our results corroborate previous studies that showed negative responses of mayflies to high water temperature (e.g., Chadwick and Feminella 2001; Alhejoj et al. 2014) and low oxygen concentrations (e.g., Nebeker 1972; Lock and Goethals 2011). Sites that were characterised by high water temperatures were also often accompanied by low oxygen content and dense aquatic vegetation. High levels of nutrients in the water support such dense growth of vegetation, leading to a decrease of oxygen level (Boeykens et al. 2017). Moreover, the decay of organic matter (especially aquatic vegetation), together with bacterial growth, animal/human metabolic activity and various synthetic sources (such as pesticides, fertilisers, pharmaceuticals, detergents) lead to elevated concentration of total organic carbon (TOC) in water (e.g., Volk et al. 2002). A part of the TOC can be explained by the increased shoreline erosion due to management and clearing of vegetation in the shoreland zone, which probably also negatively affected mayflies in this study. Riparian buffers, especially undisturbed vegetated riparian zones situated adjacent to river and streams, can greatly mitigate nutrients, sediment from surface and groundwater flow through the processes of deposition, absorption and denitrification (e.g., Peterjohn and Correll 1984). Finally, the strong negative association of mayfly assemblages with intensive agriculture in the catchment area corroborates results of previous studies that showed high mayfly sensitivity to agricultural pollution (Siegloch et al. 2014; Zedková et al. 2015). Here, as especially sensitive showed *Alainites muticus*, *Baetis lutheri* and *Oligoneuriella rhenana*, species with low and moderate tolerance to water pollution (mainly occurring in oligosaphrobic and beta-mesosaphrobic waters) (Bauernfeind et al. 2002; Mihaljević 2011). In addition, another species was distinguished as sensitive to such kind of pollution, *Baetis rhodani*. Those results could come as a surprise, as this eurytopic mayfly has a wide ecological tolerance, and generally contributes as a major part of the macroinvertebrate biomass in many European streams and rivers (Elliott et al. 1988). Nevertheless, as *Baetis rhodani* is a species complex (Williams et al. 2006), those results should be inspected in more details, using molecular analyses. Our results confirm that water pollution is one of the largest limitation factors for the majority of mayflies (Van Dijk et al. 2013; Zedková et al. 2015).

## Conclusions

This study contributes to our knowledge of mayfly relationship with environmental conditions in heavily modified and anthropogenic habitats. Various anthropogenic pressures resulted in changes in mayfly assemblage composition and structure, whereas species richness decreased. For instance, the assemblages consisted mainly of a relatively low number of widespread generalists and species characteristic for lower reaches and lentic habitats. This indicates that hydromorphological alterations could have resulted in assemblage’s “potamisation”. Moreover, highly polluted sites, with high temperatures and low oxygen content, were inhabited almost exclusively with the euryvalent *Cloeon dipterum*, or were completely unsuitable for any mayfly species, confirming the high sensitivity of mayflies to disturbances in their habitats. Our results can enable planning of management and conservation activities of lowland rivers and their biota according to the requirements of the European Water Framework Directive.
